# A Financial Incentives Program to Promote Smoking Cessation Among Recently Hospitalized Individuals: Feasibility and Acceptability Study

**DOI:** 10.2196/44979

**Published:** 2023-05-29

**Authors:** Sara Shusterman, Rodolfo Villarreal-Calderon, Adrian Gunawan, Alexis Gallardo Foreman, Charles O'Donnell, Cornelia Wakeman, Hadi Javeed, Jacob Keteyian, Jinesa Howard, Katia Bulekova, Shalen de Silva, Trevor Campbell, Karen Lasser, Hasmeena Kathuria

**Affiliations:** 1 Boston University Chobanian & Avedisian School of Medicine Boston, MA United States; 2 Vincere Health Boston, MA United States; 3 Research Computing Services, Information Services & Technology Boston University Boston, MA United States

**Keywords:** financial incentives, mobile application, smoking cessation, tobacco treatment intervention

## Abstract

**Background:**

Hospitalization is an opportunity to engage underserved individuals in tobacco treatment who may not otherwise have access to it. Tobacco treatment interventions that begin during hospitalization and continue for at least 1 postdischarge month are effective in promoting smoking cessation. However, there is low usage of postdischarge tobacco treatment services. Financial incentives for smoking cessation are an intervention in which participants receive incentives, such as cash payments or vouchers for goods, to encourage individuals to stop smoking or to reward individuals for maintaining abstinence.

**Objective:**

We sought to determine the feasibility and acceptability of a novel postdischarge financial incentive intervention that uses a smartphone application paired to measurements of exhaled carbon monoxide (CO) concentration levels to promote smoking cessation in individuals who smoke cigarettes.

**Methods:**

We collaborated with Vincere Health, Inc. to tailor their mobile application that uses facial recognition features, a portable breath test CO monitor, and smartphone technology to deliver financial incentives to a participant’s digital wallet after the completion of each CO test. The program includes 3 racks. Track 1: Noncontingent incentives for conducting CO tests. Track 2: Combination of noncontingent and contingent incentives for CO levels <10 parts per million (ppm). Track 3: Contingent incentives only for CO levels <10 ppm. After obtaining informed consent, we pilot-tested the program from September to November 2020 with a convenience sample of 33 hospitalized individuals at Boston Medical Center, a large safety-net hospital in New England. Participants received text reminders to conduct CO tests twice daily for 30 days postdischarge. We collected data on engagement, CO levels, and incentives earned. We measured feasibility and acceptability quantitatively and qualitatively at 2 and 4 weeks.

**Results:**

Seventy-six percent (25/33) completed the program and 61% (20/33) conducted at least 1 breath test each week. Seven patients had consecutive CO levels <10 ppm during the last 7 days of the program. Engagement with the financial incentive intervention as well as in-treatment abstinence was highest in Track 3 that delivered financial incentives contingent on CO levels <10 ppm. Participants reported high program satisfaction and that the intervention helped motivate smoking cessation. Participants suggested increasing program duration to at least 3 months and adding supplemental text messaging to increase motivation to stop smoking.

**Conclusions:**

Financial incentives paired to measurements of exhaled CO concentration levels is a novel smartphone-based tobacco cessation approach that is feasible and acceptable. Future studies should examine the efficacy of the intervention after it is refined to add a counseling or text-messaging component.

## Introduction

About 12.5% of US adults smoke cigarettes, with rates disproportionately higher among individuals with lower income, lower education, and mental health and substance use disorders [1,2]. Hospitalization is an opportunity to engage underserved individuals in tobacco treatment who may not otherwise have access to it [3-5]. Based on a meta-analysis of 50 randomized clinical trials, tobacco treatment interventions started in the hospital that are continued for at least 1 month post hospital discharge increase tobacco abstinence rates at 6 months post discharge [6]. Yet, there is low usage of postdischarge tobacco treatment services [7,8].

Financial incentives for smoking cessation are an intervention in which participants receive incentives, such as cash payments or vouchers for goods, to encourage individuals to stop smoking or to reward individuals for maintaining abstinence. Incentives can be delivered for participation in programs, regardless of smoking status (guaranteed or noncontingent incentives), or can be paid and scaled relative to an individual achieving or maintaining abstinence (contingent incentives). A Cochrane review found that 6-month smoking abstinence rates were higher for participants receiving financial incentives compared to controls [9].

Technology to deliver financial incentives uses a smartphone app to authenticate patient identity, a carbon monoxide (CO) monitor that connects to the smartphone to verify smoking abstinence, and a display of the incentive earned after each CO test [10,11]. Vincere Health, Inc. uses a digital wallet to store incentives earned after each test; accumulated incentives are delivered through the app based on individuals’ preferences (eg, digital check) [11]. Since this technology can be delivered in a patient’s place of residence (eg, shelter), it could overcome barriers to individuals engaging in postdischarge treatment.

Evidence supports “opt-out” approaches to offering tobacco treatment to all individuals who smoke, regardless of readiness to quit [12-18]. We therefore developed a financial incentives intervention to promote smoking cessation tailored to an individual’s preferences for contingent, noncontingent, or combination incentives. We collaborated with Vincere Health Inc. to deliver the intervention using their smartphone technology. The program was initiated during hospitalization and continued for 30 days post discharge. We report on the feasibility and acceptability of the tailored financial incentive program.

## Methods

### Preimplementation Needs Assessment

Our goal was to develop a program that was feasible and acceptable to patients, clinicians, and hospital leadership. We therefore convened an advisory stakeholder panel that included a recently hospitalized patient with tobacco dependence, clinicians, and Boston Medical Center (BMC) hospital leaders (VP of Ambulatory Operations and Professional Services and Sr. Manager of ACO Operations). Clinicians included an outpatient primary care physician-investigator with expertise in developing and implementing a financial incentive program at our safety-net hospital primary care practice (KL); a pulmonologist who directs the tobacco treatment center (HK); and tobacco-trained specialists comprised of nurse practitioners (AGF, CW), a respiratory therapist (CO), and a community health worker (JH). In the 6 months prior to implementing the financial incentive intervention, we met with stakeholders to inform intervention development.

After reviewing data with the advisory panel on the high smoking rates of 26% at BMC, demographics (largely Medicaid-insured), low engagement with postdischarge tobacco treatment [19,20], and the effectiveness of financial incentives in promoting smoking cessation [21], we concluded that adults hospitalized with tobacco dependence, particularly if low-income, could potentially benefit from such a program. BMC hospital leaders on the advisory panel recommended focusing the intervention on the 1-month postdischarge period and relayed that the total financial incentives that health care plans would likely find feasible to implement is US $50 per patient hospitalization. Coupled with limited funding for this pilot study, our research team made a pragmatic choice to develop and implement the financial incentive intervention in the 1-month postdischarge period.

At BMC, the standard of care is to offer treatment to all individuals who smoke, regardless of readiness to quit [22]. Since individuals participating in the financial incentives intervention would be in various stages of readiness to quit, we agreed that it was essential to include financial incentives tailored to an individual’s preferences for contingent (rewards based on achieving abstinence), noncontingent (rewards for participation, regardless of achieving abstinence), or combination (rewards for participation and enhanced for achieving abstinence) rewards.

### Intervention Development 

We collaborated with Vincere Health Inc. to tailor their mobile application that uses facial recognition features, a portable breath test CO monitor, and smartphone technology to instantly display and deliver financial incentives to a participant’s digital wallet after completing each CO test. At the end of the 30-day period, participants could redeem their incentives from their digital wallet through the app by choosing from 4 options (Venmo, CashApp, digital check, or digital gift card).

Individuals from Vincere Health Inc. (HJ, JK, SdS, and TC) programmed the app to encourage individuals to conduct 2 CO breath tests daily for 30 days by sending reminder SMS text messages before each scheduled test. Twice daily testing made it less plausible that participants were smoking between assessments since the half-life of CO is about 4.5 hours [23].

Incentives distributed to the digital wallet were dependent on the track chosen and the number of tests performed (maximum of 60 tests in a 30-day period). Total potential incentives earned centered around US $50, the amount deemed feasible by BMC leadership. Slightly higher incentives could be earned for demonstrating CO levels <10 parts per million (ppm); according to the Society on Nicotine and Tobacco Subcommittee on Biochemical Verification (2002), CO levels ≥8 to 10 ppm suggest recent cigarette smoking [24]. Of note, an update was published in late 2020 suggesting that an appropriate cutpoint may be 4 to 10 ppm for research and clinical purposes depending on local smoke-free legislation, smoking prevalence, and air pollution levels [25]. For this study, we chose <10 ppm because secondhand smoke exposure is higher among people with low socioeconomic status and because air pollution levels are higher in communities served by BMC.

The tracks were as follows:

- Track 1: Noncontingent, guaranteed incentives for conducting CO tests, regardless of CO levels. Participants could earn up to US $45, earning US $0.75 per test performed.
- Track 2: Combination of noncontingent and contingent incentives. Participants could earn up to US $50. For participation, participants could earn up to US $30, earning US $0.50 per test performed. Participants could earn a US $20 bonus if all 14 tests in the last 7 days demonstrated CO levels <10 ppm. No bonus was delivered if in the last 7 days any CO level was ≥10 ppm and/or if the participant conducted less than 14 tests.
- Track 3: Contingent incentives only. Participants could earn up to US $60, earning US $1 for each test with CO levels <10 ppm. No incentive was delivered for tests with CO levels ≥10 ppm.

### Ethical Considerations

Individuals were compensated up to US $50 for participation in surveys and interviews: US $10 for completing the baseline survey or interview, US $15 for completing a 2-week follow-up survey or interview, and US $25 for completing a 4-week follow-up survey or interview. Participants could additionally earn up to US $60 in financial incentive payments. Vincere Health, Inc. donated disposable CO monitors, loaner phones, and funds to cover the financial incentive payments. Research funds covered the costs of gift cards for participation in surveys and interviews. All data were linked to patients by a unique study identification number unrelated to any Health Insurance Portability and Accountability Act identifiers. Data were stored on a secure server to which only designated individuals had access, thus providing a secure environment for all project data. The Boston University Medical Center Institutional Review Board approved this study (H-39365). All procedures performed in studies involving human participants were in accordance with ethical standards of the institutional review board. Informed consent was obtained from all individual participants included in the study.

### Recruitment, Enrollment, and Onboarding

We pilot-tested the program from September to November 2020 with a convenience sample of 33 individuals who smoked cigarettes and were hospitalized at BMC, the largest safety-net hospital in New England. Although we initially planned to recruit participants starting in July 2020, based on BMC guidance on conducting research studies during the COVID-19 pandemic, we limited recruitment from September to November 2020. Participants were identified from a list of hospitalized individuals who triggered consultation to the Tobacco Treatment Consult (TTC) service based on current smoking status in the electronic health record (EHR) (20). Eligible participants were (1) ≥18 years old, (2) hospitalized at BMC, (3) able to speak and read English, (4) currently smoking cigarettes (defined as smoking at least 1 cigarette up until the day of hospital admission), and (5) able to provide informed consent. Participants were excluded if cognitively impaired or diagnosed with COVID-19. At the time of enrollment, the mobile app was only compatible with an Android operating system (OS); loaner phones were provided to individuals who did not have a mobile phone or did not have an Android OS at the time of hospitalization. Of note, by the time of publication of this study, Vincere Health, Inc. created this app for use in Apple OS (Apple iOS).

**Figure 1:**
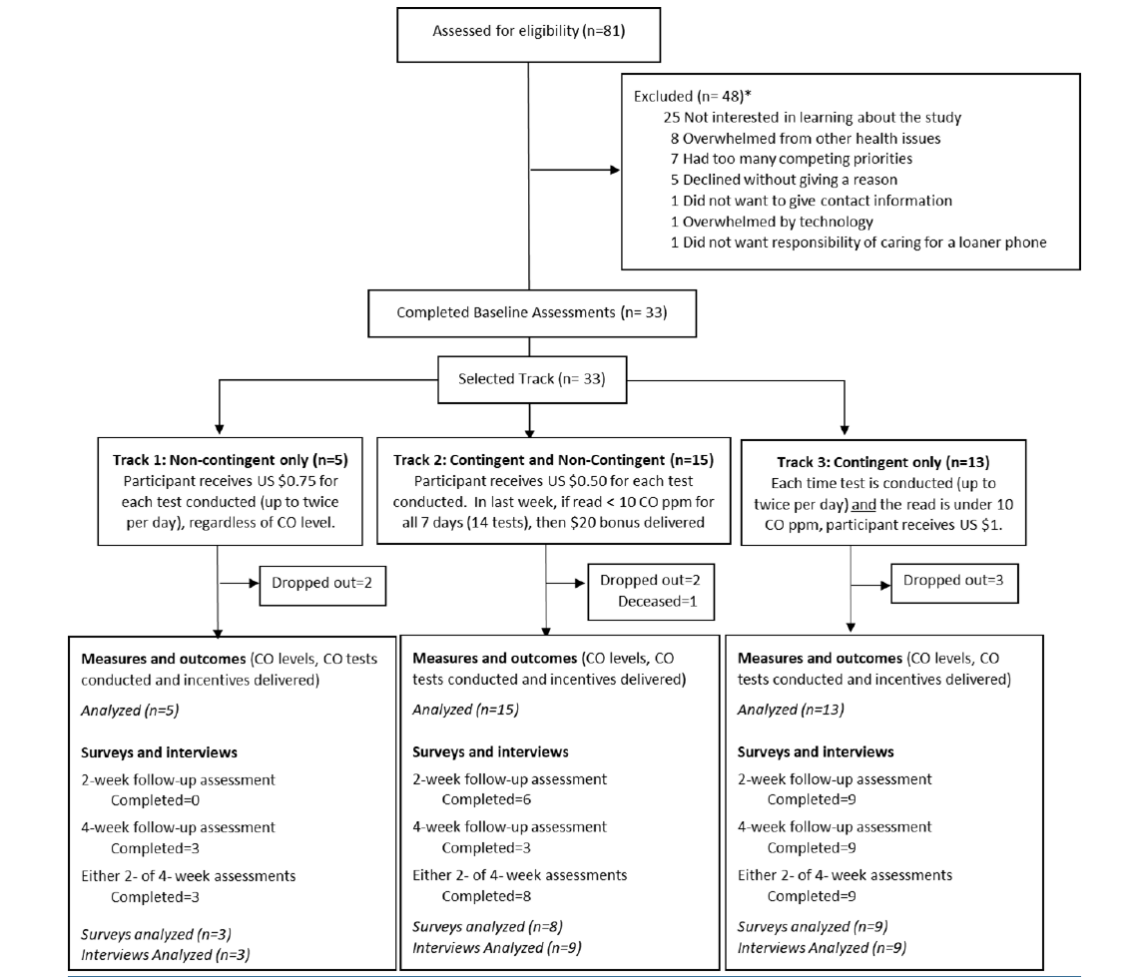
CONSORT (Consolidated Standards of Reporting Trials) flow diagram. CO: carbon monoxide.

A total of 81 individuals met screening criteria by EHR review (individuals listed as “current” for smoking status). Of the indviduals, 41% (n=33) agreed to participate; provided informed consent; chose their preference for tracks 1, 2, or 3; and enrolled. Figure 1 shows the CONSORT (Consolidated Standards of Reporting Trials) flow diagram. Per standard of care at BMC, all patients were provided bedside tobacco treatment counseling and medication recommendations, postdischarge medication recommendations to the primary inpatient team, and referral to an internal tobacco treatment clinic and/or state quitline at discharge [20].

During hospitalization, the study team gave participants the CO monitor and taught them how to use the device and the mobile app. We provided loaner smartphones to 12 participants. The onboarding process consisted of having the patient download the app, select their track choice (track 1, 2, or 3), and enter their unique 3-digit Smokerlyzer CO monitor PIN number. Prior to hospital discharge, patients were instructed to conduct their first breath test by following on-screen instructions to ensure that everything was working. Videos such as “How to do a Breath Test” were available to patients and the study team to help in the process, now updated to highlight newer features including the ability to connect wirelessly to smartphones [11]. Study staff administered baseline questionnaires in-person at the time of hospitalization and conducted 2- and 4-week questionnaires and interviews by telephone.

### Quantitative Assessments

At the time of enrollment, we collected baseline demographics and smoking characteristics. Engagement was assessed by how frequently participants conducted CO tests. Program satisfaction was measured at 2- and 4-week questionnaires, thus limiting recall bias. Overall program satisfaction was rated on a scale of 1 to 7 (range: 1=not satisfied at all to 7=very much satisfied). The 2- and 4-week questionnaires additionally assessed the perceived impact of the program on motivation to stop smoking.

### Qualitative Assessments

We qualitatively measured feasibility and acceptability at both 2 and 4 weeks through semistructured interviews. The interviews assessed participants’ (1) perceived impact of the intervention in motivating smoking cessation, (2) experiences with the program, and (3) suggestions for improvement. Interviews were audio-recorded and transcribed verbatim.

### Data Analyses

Descriptive statistics were calculated using the SPSS v18 (IBM Corp) and R (R Foundation for Statistical Computing) statistical programming software. For qualitative interviews, we used inductive content analysis to analyze transcripts and performed unstructured coding of transcripts to identify themes. Three members (SS, RVC, and HK) developed a codebook and independently reviewed all transcripts and added codes until the team reached consensus. We finalized conceptual categories, grouped themes in each category, and identified quotes best highlighting themes.

## Results

### Baseline Survey Data

The median age of participants was 46 (range 30-74) years. Participants were 42% (14/33) female and 55% (18/33) non-Hispanic Black American (Table 1). Individuals smoked 6.8 (SD 4.7) cigarettes daily.

**Table 1:** Baseline characteristics of sample.

Characteristics of patients		All participants (N=33)	Track 1: noncontingent only (n=5)	Track 2: contingent and noncontingent (n=15)	Track 3: contingent only (n=13)
**Baseline demographics**					
	Age (years), median (range)	46 (30-74)	47 (41-62)	46 (31-63)	46 (30-76)
	Female, n (%)	14 (42)	1 (20)	7 (47)	6 (46)
**Race/ethnicity, n (%)**					
	Non-Hispanic White	9 (27)	0 (0)	7 (47)	2 (15)
	Non-Hispanic Black	18 (55)	3 (60)	5 (33)	10 (77)
	Hispanic (any race)	5 (15)	2 (40)	2 (1)	1 (1)
	Other	1 (3)	0 (0)	1 (7)	0 (0)
Income < US $15,000, n (%)		18 (55)	3 (60)	10 (67)	5 (38)
Less than high-school education, n (%)		22 (67)	2 (40)	12 (80)	8 (62)
**Smoking characteristics**					
	Years smoked, median (range)	24 (7-45)	21 (7-32)	21 (10-45)	25 (15-44)
	Average pack-year (SD)	24 (11)	21 (9)	23 (9)	27 (14)
	Cigarettes smoked per day, mean (SD)	6.8 (4.7)	11.9 (5.8)	8.6 (7.7)	9.9 (6.6)
	Very important/important to quit, n (%)	23 (70)	4 (80)	10 (67)	9 (69)
	Very high/high motivation to quit, n (%)	22 (67)	3 (60)	8 (53)	11 (85)
	Ready to quit smoking in 30 days, n (%)	13 (39)	1 (20)	4 (27)	8 (62)
**Primary discharge diagnosis, n (%)**					
	Any smoking-related disease^a^	13 (39)	2 (40)	8 (53)	3 (23)
	Cardiovascular diseases	8 (24)	2 (40)	5 (33)	1 (8)
	Respiratory diseases	3 (9)	—^b^	1 (7)	2 (15)
	Neoplasms	1 (3)	—	1 (7)	—
	Perinatal conditions	1 (3)	—	1 (7)	—

^a^Smoking-related diseases: cardiovascular (peripheral vascular, coronary heart disease, stroke), respiratory (chronic obstructive pulmonary disease, pneumonia), neoplasms, and perinatal conditions as specified in the Surgeon General’s report [26].

^b^Not available.

### Outcomes and Measures

Of the 33 participants, 15% (n=5) enrolled in track 1, 45% (n=15) in track 2, and 27% (n=13) in track 3 (Figure 1). Overall, 61% (n=20) of participants engaged with the program, as measured by conducting at least 1 CO test each week. The program was discontinued by 30% (n=10) of participants within 24 hours of hospital discharge. On the first day after hospital discharge, the mean CO levels were 11.9 (range 2.6-36.1) ppm. The average incentive earned per participant was US $14.19. Seven patients had consecutive CO levels <10 ppm during the last 7 days of the program; 4 of these patients were enrolled in track 3. Table 2 shows data by enrolled track.

**Table 2:** Outcomes and measures, stratified by track.

Outcomes and measures	Track 1: noncontingent only (n=5)	Track 2: contingent and noncontingent (n=15)	Track 3: contingent only (n=13)
Engagement (conducting ≥1 CO^a^ test each week), n (%)	3 (60)	8 (53)	9 (69)
Tests conducted each week^b^, mean (range)	5.6 (1.5-12.5)	6.2 (1-14)	9.4 (0.8-14)
CO levels (ppm; first-day posthospital discharge), mean (range)	25.3 (14.5-36.1)	13.4 (5.3-28.1)	6.4 (2.6-10.1)
Patients with CO levels <10 ppm in last 7 days of program, n (%)	1 (20)	2 (13)	4 (31)
Total incentives delivered per participant (US $), mean (range)	10.60 (1-45)	13.02 (0.75-45)	20.31 (1-60)

^a^CO: carbon monoxide.

^b^The number of CO tests conducted to calculate the average CO levels: track 1: 66 total CO tests; track 2: 211 total CO tests; track 3: 174 total CO tests.

### Perceptions of Program and Smoking Behavior

Of the 33 participants, 61% (n=20) completed either the 2- or 4-week questionnaires. Responses were similar for 2- and 4-week data; for individuals who completed both 2- and 4-week questionnaires, we only analyzed 4-week responses (Figure 1). We assessed end-of-program smoking behavior at 4 weeks by self-report (n=15). Of these participants, 87% (n=13) reported stopping smoking or smoking less. The 3 participants who reported stopping smoking were in track 3.

Of 20 participants, 90% (n=18) of participants were (1) very likely or likely to recommend the program to others and (2) strongly agreed or agreed with the statement “the program is making me think about quitting smoking,” and 95% (n=19) rated the program very highly or highly. All participants wanted to extend the program beyond 30 days. Table 3 details responses on satisfaction and likability of the program, stratified by enrolled track.

**Table 3:** Patient satisfaction, stratified by track.

	All participants (N=20), mean (SD)	Track 1: noncontingent only (n=3), mean (SD)	Track 2: contingent and noncontingent (n=8), mean (SD)	Track 3: contingent only (n=9), mean (SD)
Overall satisfaction (7-point scale^a^)	6.35 (0.91)	5.67 (0.94)	6.13 (1.05)	6.78 (0.42)
The degree to which the program motivated smoking cessation (7-point scale)	6.21 (0.97)	6.67 (0.47)	5.75 (0.97)	6.25 (0.97)
The degree to which participants thought program would be helpful to family/friends (7-point scale)	5.9 (1.4)	5.67 (1.25)	5.86 (1.36)	6.1 (0.93)

^a^Likert 7-point scale (range: 1=not satisfied at all to 7=very much satisfied).

### Qualitative Data

Of the 33 participants, 61% (n=20) participated in the 2- and/or 4-week interviews; we analyzed both 2- and 4-week interviews (Figure 1). Supporting quotes are identified by patient number, enrolled track, and interview week.

#### Engagement and Interactions With the Program

Participants said they frequently engaged with the program and enjoyed the interactions:

My favorite part was seeing my progress. That was the best part because it (CO levels) always came back really good, so that excited me to stay with it.

P12, Track 3, 4-week

For participants with low engagement, when probed for the reasons why, they responded that situations such as being rehospitalized sometimes precluded the ability to take CO tests:

Sometimes, the timing with my schedule, with my therapy and everything, and then at night, I’d be tired. I would be sleeping by the time it’s time to do it.

P17, Track 2, 4-week

Another barrier to engagement was not having Wi-Fi, particularly for individuals who experienced difficulty paying cellular bills, thus precluding them from performing CO tests*.*

Thanks to the Wi-Fi at the rehab, I was able to continue doing my test each day. They discharged me early to go home. I hadn’t paid my cellphone bill, and they shut off my service. There’s no Wi-Fi there that I could access. I was ‘Cut-off from the world,’ including being able to take the test.

P20, Track 3, 4-week

#### Program Satisfaction

##### Technical Aspects

Participants were satisfied with the program, largely because they found it easy to use

I didn't find any problems at all. … it was kind of self-explanatory. Instructions would pop up on the screen. “Please plug device into phone,” so I did that. From there, each step was very easy to maneuver.

P20, Track 3, 4-week

Some participants, however, experienced technical issues with Wi-Fi:

At one point the Wi-Fi and the Bluetooth weren’t connecting.

P31, Track 3, 2-week

And facial recognition features:

I had a beard when I was in the hospital, and I shaved it to a goatee, and the facial recognition, sometimes, it takes a little longer now.

P30, Track 2, 2-week

Individuals appreciated the responsiveness and guidance by technical staff as problems arose:

Because if I didn’t know what I was doing, then I could call. At least I had some type of assistance to guide me, to let me know how to use the device itself, or walk me through the steps of how to use it

P21, Track 2, 2-week

##### Text Reminders to Blow Into CO Device

Participants thought the reminders to blow into the device were helpful:

Because sometimes, I would forget, or I’d be out and about, and I would get a reminder, so it was very good for me.

P12, Track 3, 4-week

Others thought the reminders were too frequent:

It was definitely repetitive at some points.

P5, Track 1, 4-week

#### Perceptions of the Program

##### Perceived Impact of the Program on Motivation to Stop Smoking

Several participants discussed how they cut down or stopped smoking while enrolled in the program:

Well, I’m completely cut down. I can say with pride and throwing my chest out there, I’m smoke free. I am now a former smoker.

P20, Track 3, 4-week

Individuals indicated that the program itself motivated smoking cessation:

It was relevant. I really like it. It’s a new way of getting more people motivated to stop smoking

P32, Track 1, 4-week

Some described how health issues were the reason for enrolling in the program:

I was just newly diagnosed with cancer, and that’s what motivated me as well to want to be a part of this program.

P12, Track 3, 4-week

##### The Financial Incentives Itself Increased Motivation to Stop Smoking

Participants described how the incentives helped with financial hardships:

I have a financial hardship, so it’s helping me with my financial hardship. I’m not gonna lie. I’m not trying to be selfish or like it’s all about the money, but it’s helping me be able to do things that I need to do or buy the things that I need to buy.

P8, Track 3, 2-week

Some discussed that while the incentive provided the initial motivation to stop smoking and/or enroll in the program, it also provided intrinsic motivation to stop smoking:

First, it is the money. I’m not gonna lie, but then after a while, it’s more of actually wanting to do it for myself

P21, Track 2, 2-weeks

##### CO Monitoring Motivated Cessation

Many stated that a large driver of increased motivation to stop smoking was the CO readout:

With that little thing with the carbon monoxide, it makes you don’t wanna smoke no more seeing that. It makes you think about how much damage you’re doing to yourself by smoking

P17, Track 2, 2-weeks

#### Suggestions for Improvement

Suggestions for improvement ranged from providing additional support (eg, supportive phone calls and/or text messages) to suggestions regarding timing and delivery of incentives.

- Providing supportive phone calls when needed: “I think it would be helpful for anybody who was really struggling to have somebody to talk to about it, just like an A.A. meeting.” (P5, Track 1, 4-week)
- Adding a text-message component: “It (text messages) would be helpful because you’re getting a tip about quitting and the health problems that come with smoking and all that.”(*P* 17, Track 2, 4-week)
- Increasing length of incentives to at least 3 months: “Oh, like three months… A month isn't a whole lot, especially for someone who's really trying to quit.” (P15, Track 2, 4-week)
- Increasing the incentive amount: “It could have been a little more but it was alright, maybe another $50” (P33, Track 3, 4-week)
- Increasing flexibility in timing of CO testing: “If you miss one, you can make up with the third one if it’s at a different time.” (P17, Track 2, 4-week)
- Expanding the inclusion criteria to all individuals who smoke, not just hospitalized individuals: “I feel that everybody should have the opportunity to be a part of that program. Not just you have to be inpatient when you start it. Post flyers around the hospital and even outside the hospital. I think that would be really good because there’s people that want to quit smoking, especially now with this COVID‑19 going on.” (P12, Track3, 4-week)

## Discussion

We provide evidence of the feasibility and acceptability of delivering financial incentives to promote smoking cessation among recently hospitalized individuals. Our intervention is unique because it delivers incentives tailored to individuals’ preferences for contingent, noncontingent for participation, or a combination scheme, which is an important feature given that this study is inclusive of all hospitalized individuals who smoke, regardless of readiness to stop smoking.

Participants reported high satisfaction with the intervention and that it motivated smoking cessation. In qualitative interviews, individuals reported that the program made them think about stopping smoking, regardless of the track chosen. Several individuals reported that seeing their CO readouts enabled them to understand their smoking behavior and related health effects, and encouraged them to stop smoking. Previous studies have shown that CO readouts are a valuable monitoring and feedback component of tobacco treatment programs [27], and recent studies suggest that personal use of CO monitors is acceptable and motivating in promoting smoking cessation [28]. Of 15 particiaptns, 87% (n=13) of participants reported stopping smoking or smoking less at the end of the program. Our results show that engagement with the financial incentive intervention as well as in-treatment smoking behavior change was highest in the track that delivered financial incentives contingent on abstinence, which will be the focus of further app development and future studies.

Participants also described their financial hardships; the prospect of receiving money to stop smoking motivated abstinence. Two previous studies have demonstrated the effectiveness of financial incentives in promoting smoking cessation in safety-net settings: Kendzor et al [29] included individuals who attended a tobacco cessation clinic and Lasser et al [21] included individuals in the primary care setting who were in the contemplation or preparation stage of readiness to quit smoking but not already in treatment. A recent study demonstrated the feasibility of an automated smartphone-based approach to delivering financial incentives for smoking cessation to socioeconomically disadvantaged adults willing to stop smoking within 7 days [10]. Our study adds to the growing evidence that an automated smartphone-based approach to delivering financial incentives is a feasible and acceptable behavioral intervention for recently hospitalized individuals who smoke, even among low-income populations who are not ready to quit.

Several technical and program features facilitated participation. Participants described the ease of use, the text reminders to blow into the device, and the availability of technical support staff were particularly helpful. Patients also described technical barriers such as Wi-Fi issues and problems with facial recognition.

Participants made suggestions for improvement. Some suggested increasing the incentive amount. While incentives amounts have varied across studies, a Cochrane review showed no significant difference between trials paying less than US $100 compared to those paying more than US $700 [9].

Many individuals also discussed how increasing the intervention duration to 3 months would enhance the program. A recent study suggests that a longer duration of postdischarge tobacco treatment of at least 3 months may be needed to sustain long-term tobacco abstinence. Individuals also suggested pairing the program with supportive calls and/or supplemental SMS text messaging [30]. A previous study by Lasser and colleagues [21] demonstrated that a multicomponent intervention consisting of patient navigation and financial incentives for smoking cessation in the ambulatory setting significantly increased cessation rates. A pilot randomized controlled trial testing the efficacy of a smartphone app that provides exhaled CO readings with message support showed high satisfaction, but no differences in smoking cessation [31]. Vincere Health, Inc. has since expanded features of the financial incentives app to include 2-way SMS and supportive calls from tobacco treatment specialists and behavioral health coaches.

As suggested by our data, we plan to refine the financial incentive intervention by increasing program duration to at least 3 months and adding supplemental SMS text messaging (eg, content adapted from the National Cancer Institute’s Smokefree TXT [32] and from prior work by our study team [33]) and/or coaching to promote smoking cessation. In addition to testing the efficacy of the intervention on maintaining smoking abstinence achieved during hospitalization, in future studies, we will test whether the intervention motivates smoking abstinence in those currently smoking in the ambulatory setting.

Our study has strengths and limitations. A strength is that we included individuals regardless of readiness to quit, an important inclusion since “opt-out” approaches can increase smoking cessation [12-18]. We conducted assessments both during and right after study completion, thus minimizing recall bias. Given the relatively short half-life of CO [23], twice daily testing is an important feature of the app. However, few patients tested twice daily as intended, making it plausible that participants were smoking between assessments. Our small sample size from a single site limits generalizability and may not reflect perspectives of all individuals. While our underserved population is a strength, it created a limitation for assessing feasibility and acceptability: we were unable to reach 40% of participants by phone at study end, a finding reported in other mobile-based interventions in underserved populations [34-36]. Although we analyzed feasibility to program implementation from the perspective of the patient (eg, ease of use), we did not assess implementation feasibility from the perspective of insurers, hospital system, and technical support (eg, predicted cost, resource availability). Feasibility from these other perspectives through surveys and interviews will be important implementation outcomes to assess in future studies.

### Conclusions

Financial incentives paired to measurements of exhaled CO concentration levels is a novel smartphone-based tobacco cessation approach that is feasible and acceptable. Future studies should examine the efficacy of the intervention after it is refined to add a counseling and/or SMS text messaging component.

## Abbreviations

Apple iOS: Apple OS
BMC: Boston Medical Center
CO: carbon monoxide
CONSORT: Consolidted Standards of Reporting Trials
EHR: electronic health record
OS: operating system
ppm: parts per million
TTC: tobacco treatment consult

## Data Availability

The data collected and analyzed during this study are available from the corresponding author on reasonable request.
